# Study on the Prognostic Values of TTC36 Correlated with Immune Infiltrates and Its Methylation in Hepatocellular Carcinoma

**DOI:** 10.1155/2022/7267131

**Published:** 2022-07-08

**Authors:** Wei Jing, Ruoyu Peng, Xiaogai Li, Shaogang Lv, Yu Duan, Shitao Jiang

**Affiliations:** ^1^Department of Clinical Laboratory, The First Affiliated Hospital of Zhengzhou University, Key Laboratory of Laboratory Medicine of Henan, Zhengzhou 450000, China; ^2^Department of Nuclear Medicine, The First Affiliated Hospital of Zhengzhou University, Henan Medical Key Laboratory of Molecular Imaging, Zhengzhou 450000, China

## Abstract

Hepatocellular carcinoma (HCC) remains an incurable disease with a very poor clinical outcome. The purpose of this article was to investigate whether the expression or methylation of tetrapeptide repeat domain 36 (TTC36) could be used as a prognostic marker in hepatocellular carcinoma. TCGA database was used to obtain information on HCC gene expression and the associated clinical features of HCC patients. Differentially expressed genes (DEGs) were screened between 374 HCC specimens and 50 nontumor specimens. The expression and prognostic value of TTC36 were analyzed. The correlations between TTC36 and cancer immune infiltrates were investigated via TIMER. In this study, HCC specimens and nontumor specimens were compared and 35 DEGs were found between them. Among the 35 DEGs, the expression of TTC36 was significantly reduced in HCC samples compared with nontumor samples. Survival tests revealed that patients with low TTC36 expression had a shorter overall survival than patients with high TTC36 expression. TTC36 was found to be an independent predictive factor for HCC in both univariate and multivariate regression analyses. TTC36 was negatively regulated by TTC36 methylation, leading to its low expression in HCC tissues. Immune analysis revealed that TTC36 expression has significant correlations with B cell, T cell CD4+, neutrophil, macrophage, and myeloid dendritic cell. Finally, TTC36 expression was dramatically reduced in HCC cells, and overexpression greatly suppressed HCC cell proliferation and invasion, according to our experimental results. Overall, our data suggested that TTC36 could be applied as a prognostic marker for predicting outcome and immune infiltration in HCC.

## 1. Introduction

Hepatocellular carcinoma (HCC) is one of the most common malignancies and the third leading cause of tumor-related death, with more than 620,000 new cases diagnosed each year [1]. The majority of all cases can be attributed to the population of people living in Asia and Africa. HCC is characterized by a gradual beginning, a high level of aggressiveness, and a rapid rate of progression [2]. HBV infection is still regarded to be the primary cause of HCC worldwide, and between 50 and 80 percent of cases of HCC are linked to HBV [3, 4]. The prognosis for HCC patients has been substantially improved thanks to surgical and chemotherapeutic improvements; however, those who have been identified with advanced HCC have not received appropriate treatment options [5, 6]. Metastasis is a major factor in the poor prognosis and unsuccessful therapy of individuals with HCC [7]. Thus, there is still an immediate need to find more critical driving oncogenes, particularly those that change the makeup of the immune microenvironment in HCC.

Prior developments in high-throughput systems for profiling genome-wide alterations have greatly improved the genomic and molecular characterization and therapy of HCC [8]. For instance, Hou et al. reported that the prognosis of patients with high levels of SKA3 expression in their HCC is poor. Tumor growth in HCC is accelerated by increased SKA3 expression, which controls CDK2/P53 phosphorylation [9]. Che and his group showed that the expressions of ALKBH5 were found to be associated with a poorer outcome in patients with HCC whose tumors had ALKBH5 downregulation. Functionally, ALKBH5 inhibited HCC cell proliferation and invasion via LYPD1 epigenetic inhibition directed by m6A in vitro and in vivo [10]. According to these findings, potential diagnostics and therapeutic targets for HCC patients could be derived from certain functional genes.

An enzyme known as TTC36, also known as HBP21 (Hsp70-binding protein 21), has rarely been described in the previous few decades. This protein encodes three adjacent TRP repeats [11, 12]. In the current study, TTC36 expression and DNA methylation in HCC were examined. Using data from TCGA datasets; we then assessed the predictive significance of TTC36 in HCC patients. In addition, we investigated whether or not there was a connection between TTC36 and the presence of immune cells in the immunological microenvironment by using the tumor immune estimate resource (TIMER). Finally, we performed gain-of-function experiments to explore the function of TTC36 on HCC growth. HCC patients with high levels of TTC36 had a better prognosis, according to this study's findings. Moreover, we found evidence of a possible connection between TTC36 and immune cells in HCC as well as the biological processes connected with TTC36.

## 2. Materials and Methods

### 2.1. Data Source

The mRNA expression patterns of the patients with HCC were collected from TCGA, which was calculated using an Illumina HiSeq RNA-seq platform, which had 374 HCC samples and 50 neighboring nontumor samples as of July 6, 2021. Then, using the cBioPortal website, we retrieved the methylation profiles of patients from TCGA datasets. The local ethics committees did not need to accept this research because the data from TCGA are open-access and available to the general public.

### 2.2. Differentially Expressed Genes (DEGs) between HCC and Nontumor Samples

In the first step, we retrieved TCGA database's raw count of the HCC mRNA expression patterns. Over 57,000 profiles of the expression of various mRNAs were included in the HCC RNA sequencing data. Then, using the DESeq R program, the differentially expressed genes (DEGs) were computed, following investigation focused on the dataset's DEGs with absolute log_2_ fold change (FC) more than three and an adjusted *P* value less than 0.01.

### 2.3. Correlation Analysis of Immune Cell Infiltration

TIMER is a user-friendly web interface (http://cistrome.shinyapps.io/timer) and consists of six functional modules, including the association of tumor-infiltrating immune cells (TIICs) abundance with gene expression (Gene), DNA somatic copy number alterations (SCNA), somatic mutations (Mutation), and overall survival (Survival) as well as analysis of differential gene expression (DiffExp) and gene-gene correlations (Correlation) [13]. TIMER was utilized to examine the association between TTC36 and tumor purity, as well as numerous immunocytes, including CD4+ T cells, macrophages, neutrophils, B cells, and CD8+ T cells. Studying the relationships between TTC36 and immune cell infiltration was carried out by the use of Spearman correlation analysis.

### 2.4. Functional Enrichment Analysis

In the initial analysis of TCGA datasets, patients with HCC were categorized into high and low TTC36 expression groups. Genes with a false discovery rate of less than 0.05 were selected for further study. Gene terms with ∣logFC | ≥3 combined with *P* value less than 0.05 were viewed as significant. The “clusterProfiler” and DOSE packages in R were utilized in order to conduct disease ontology (DO) enrichment analysis on genes whose expression levels were significantly different [14]. Then, in order to investigate the functional roles of TTC36 in HCC, we decided to use KEGG enrichment analysis.

### 2.5. Cell Culture and Reagents

The human HCC cell lines HepG2, Hep3B, Huh7, and HCCLM3 and the normal cell line LO-2 were purchased from the Type Culture Collection Committee of the Chinese Academy of Sciences (Shanghai, China). Cultured in DMEM (HyClone) with penicillin and streptomycin and 10% fetal bovine serum (Gibco) at 37°C in 5% CO_2_ at 37°C, the cells were harvested.

### 2.6. Lentivirus Transfection

Using the plasmid we purchased, we were able to reassemble the overexpression vector. Genomeditech Biotechnology Co., Ltd. provided the lentiviral vector system. The culture media was changed two days after infection, and 2 g/mL puromycin (Solarbio, Shanghai, China) was added to the medium for screening purposes.

### 2.7. Reverse Transcription-Quantitative Polymerase Chain Reaction (RT-qPCR)

TRIzol (Invitrogen, China) was applied to extract total RNA from HCC cells in accordance with the manufacturer's procedure. RNA purity and quantification were examined by the use of the UV/VIS spectrophotometer (Alpha 1500, China). Based on the manufacturer's instructions, RT was used to synthesize cDNA (Thermo Scientific, Inc.) for qPCR analysis. RT-qPCR was applied to detect the relative expressions of TTC36, and the GAPDH gene was applied as an internal reference. Denaturation at 95°C for 5 minutes was followed by 40 cycles of denaturation at 95°C for 10 seconds, annealing at 60°C for 30 seconds, and extension at 72°C for 30 seconds. The sequences of the primers were as follows: TTC36 forward 5′-TTGGAGACATTGTTGGATTGGAC-3′ and reverse 5′-CACGGTTGTTGTAGGCTGAAG-3′; GAPDH forward 5′-CTGGGCTACACTGAGCACC-3′ and 5′-AAGTGGTCGTTGAGGGCAATG-3′.

### 2.8. Cell Proliferation Test

It was determined that CCK-8 could be used to determine the viability of Huh7 and Hep3B cell lines 48 hours after transfection. The cells were then diluted to 3 × 10^6^/mL and plated in 96-well plates with 3000 cells/well for growth in normal conditions. After 48 hours of culture, 10 L of CCK-8 solution (10 L, Sigma, China) was added to the incubator at 37°C and 5% CO_2_ for an additional 2 hours of culture. The OD value at 450 nm was measured to determine cell proliferation and to plot growth curves using a microplate reader (Bio-Rad, Cal, USA). There were three separate runs of the experiment.

### 2.9. Transwell Assay

Transwell invasion experiments were carried out using Corning-Costar transwell chambers with a Matrigel coating. The pore size of the chambers was 8 micrometers (Sigma). In the top compartment, we introduced transfected HCC cells that had been previously resuspended in medium that did not contain FBS. In the bottom chamber, we filled it with medium that contained 10% FBS as a chemotaxin. Following a period of 48 hours, the cells were fixed and stained. Cells were selected at random from five different fields, counted, and photographed using a microscope.

### 2.10. Statistical Analysis

The R software (version 3.6.1, R Core Team, Massachusetts, USA) and GraphPad Prism version 8.0 (GraphPad, La Jolla CA, USA) were used for all analyses in the present study. To compare the two groups, we used unpaired Student's *t*-test. We used the Kaplan-Meier curve and the log-rank test to examine the survival differences between two groups. HCC patients' prognostic variables were also identified using univariate and multivariate assays. *P* < 0.05 was defined as statistically significant.

## 3. Results

### 3.1. Identification of DEGs in HCC

**Figure 1:**
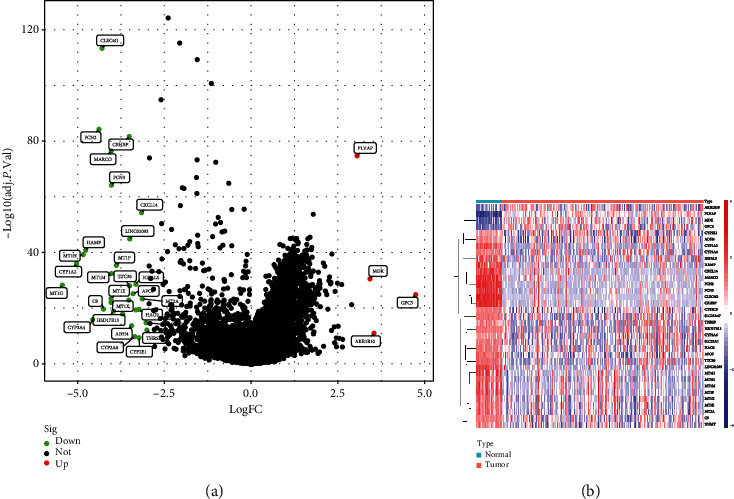
DEGs between HCC and normal liver specimens. (a) The volcano plot from TCGA datasets for the DEGs. High expression is shown by the color red; low expression is indicated by the color green. (b) Heatmap of differentially expressed genes.

To screen the DEGs in HCC, we downloaded the HCC dataset from TCGA datasets, and there were a total of 374 HCC tissues and 50 normal liver specimens analyzed in this study. These microarray data were processed, and the DEGs were discovered using the R program. Based on the R analysis, 35 DEGs met our selection criteria (an adjusted *P* value of <0.01, fold change ≥ 3.0), including 4 upregulated and 32 downregulated genes (Figures 1(a) and 1(b)).

### 3.2. The Expression and Clinical Significance of TTC36 in HCC Patients

**Figure 2:**
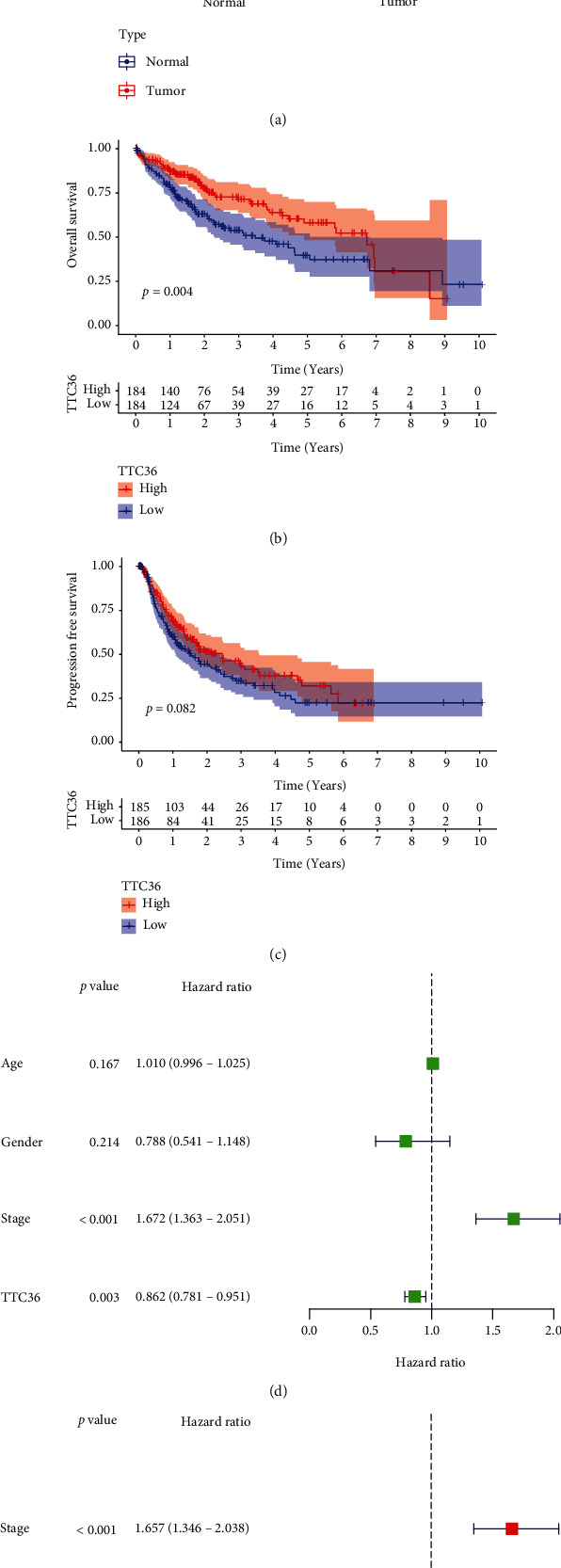
TTC36 was lowly expressed in HCC and predicted a favorable outcome. (a) The expression of TTC36 between normal and HCC tissues. (b, c) Comparing survival curves with high and low TTC36 expression using Kaplan-Meier methods. (d, e) Univariate and multivariate analysis was applied to determine the prognostic values of TTC36 expression in HCC patients.

Among the 35 DEGs, our attention focused on TTC36 whose function in tumors was rarely reported. As exhibited in Figure 2(a), the expressions of TTC36 were distinctly decreased in HCC specimens compared with normal specimens. Survival assays suggested that patients with low TTC36 expressions showed a shorter overall survival than those with high TTC36 expressions (Figure 2(b), *P* = 0.004). Although we did not observe a distinction between the PFS of patients with high TTC36 expression and the PFS of patients with low TTC36 expression, a distinct trend that low TTC36 expression was associated with a shorter progression-free survival can be observed (Figure 2(c), *P* = 0.082). In the univariate survival analysis, clinical stage (HR = 1.672, *P* < 0.001) and TTC36 expression (HR = 0.781, *P* = 0.003) were significant adverse prognostic factors for overall survival (Figure 2(d)). More importantly, multivariate analysis results revealed that clinical stage (HR = 1.657, *P* < 0.001) and TTC36 expression (HR = 0.877, *P* = 0.007) are independent prognostic factors for overall survival (Figure 2(e)).

### 3.3. A Study of the Relationship between DNA Methylation and TTC36's Survival

**Figure 3:**
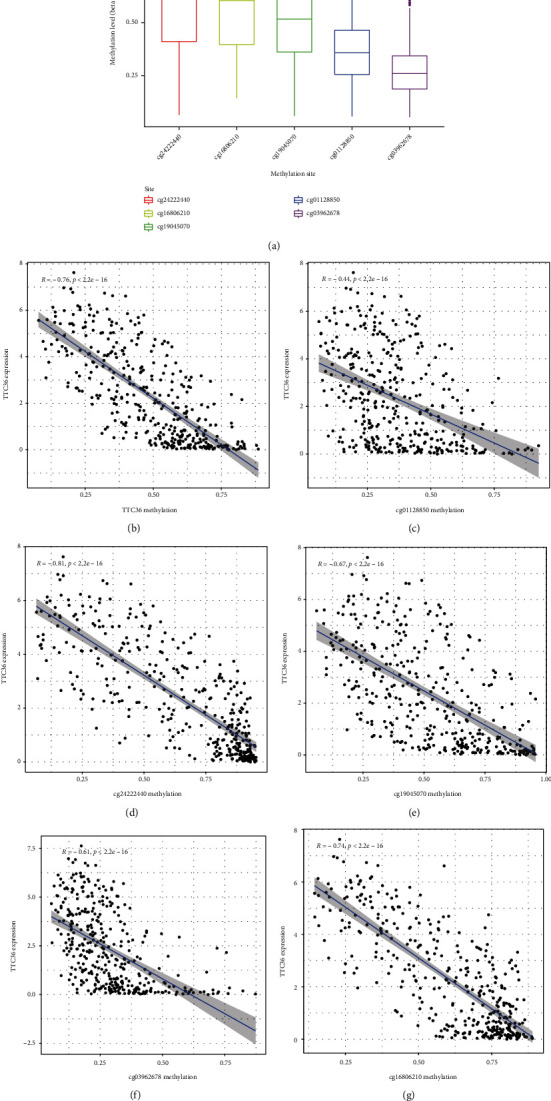
A number of sites' methylation levels were shown to be associated with TTC36 expression. (a) Methylation histograms at five different methylation locations. (b) TTC36 DNA methylation significantly impacted the expression of TTC36. Correlation assays of TTC36 with the methylation of (c) cg01128850, (d) cg24222440, (e) cg19045070, (f) cg03962678, and (g) cg16806210.

**Figure 4:**
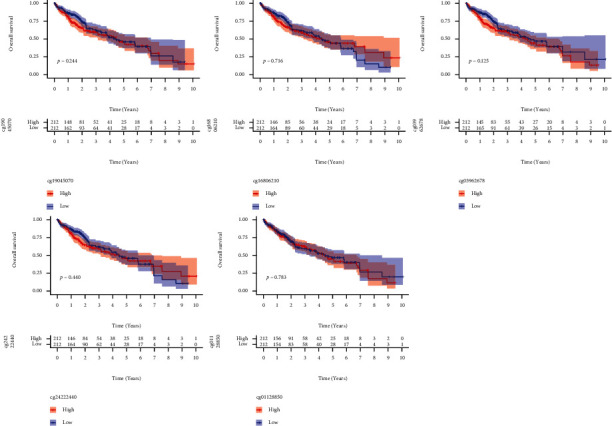
Kaplan-Meier curves for overall survival of low and high TTC36 DNA promoter CpG sites in HCC patients, including cg01128850, cg24222440, cg19045070, cg03962678, and cg16806210.

**Figure 5:**
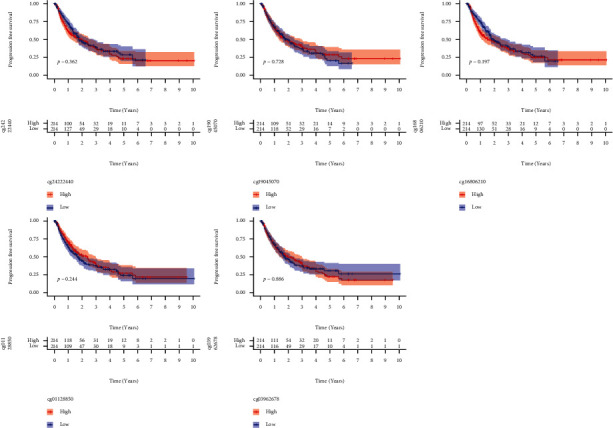
Kaplan-Meier curves for progression-free survival of low and high TTC36 DNA promoter CpG sites in HCC patients, including cg01128850, cg24222440, cg19045070, cg03962678, and cg16806210.

**Figure 6:**
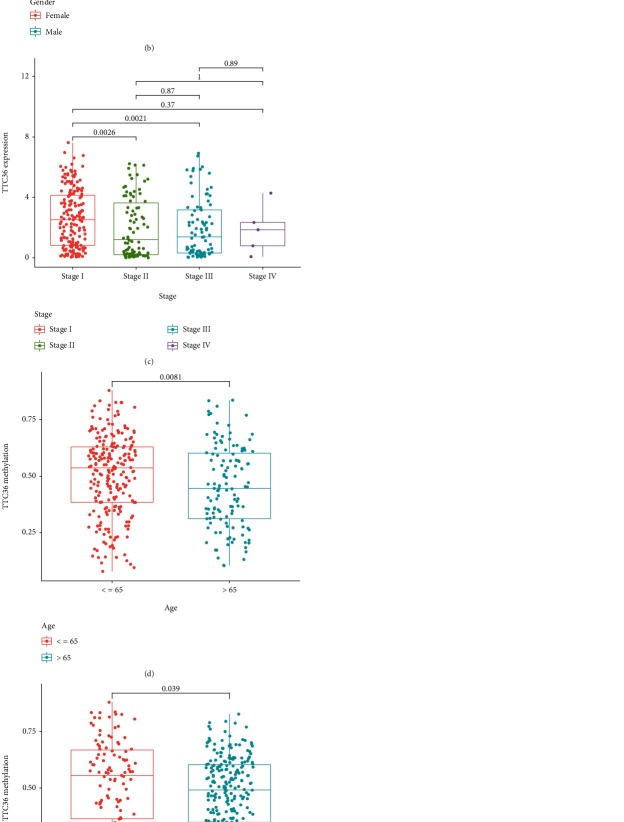
Clinical and pathological aspects of TTC36 expression/methylation in TCGA datasets. (a) Age and TTC36 expression. (b) Gender and TTC36 expressions. (c) Clinical stage and TTC36 expressions. (d) Age and TTC36 methylation. (e) Gender and TTC36 methylation. (f) Clinical stage and TTC36 methylation.

Then, we looked at TTC36's methylation level.  Figure 3(a) clearly shows the distribution of the five TTC36 CpG sites. Besides, a correlation between TTC36 expression and TTC36 DNA methylation was also found to be strongly negative (Figure 3(b)). TTC36 CpG sites that were methylated most heavily were identified by using Pearson correlation analysis. We observed that methylation of cg01128850, cg03962678, cg16806210, cg19045070, and cg24222440 was negatively related to the expression of TTC36 (Figures 3(c)–3(g)). On the other hand, all CpG sites were not associated with overall survival when we used Kaplan-Meier methods to investigate their predictive value for methylation at CpG sites (Figure 4) and progression-free survival (Figure 5) of HCC patients. Then, a broader range of clinical variables was examined in relation to TTC36 expressions. Our group found that low TTC36 expression was related to gender and clinical stage, but not associated with age (Figures 6(a)–6(c)). In addition, we found that TTC36 DNA methylation was associated with gender, but not associated with age and clinical stage (Figures 6(d)–6(f)).

### 3.4. Functional Correlation Analysis

**Figure 7:**
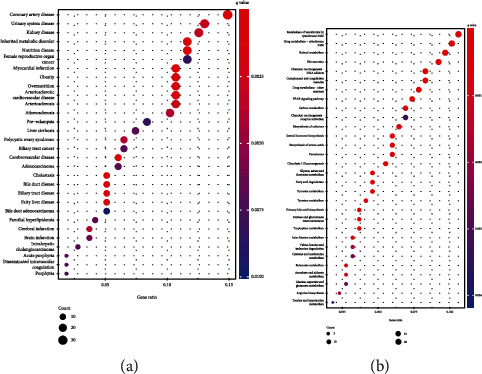
Enrichment analysis of disease ontologies and KEGG assays are used to uncover possible biological processes. (a) Disease ontology enrichment analysis of DEGs. (b) KEGG assays of DEGs.

To explore the possible roles of TTC36 in HCC progression, we screened 351 genes which were associated with the expression of TTC36. Then, we used DO pathway enrichment analyses which revealed that diseases enriched by DEGs were mainly associated with coronary artery disease, urinary system disease, kidney disease, inherited metabolic disorder, liver cirrhosis, and adenocarcinoma (Figure 7(a)). Moreover, the results of KEGG analysis indicated that the 351 genes were mainly enriched in metabolism of xenobiotics by cytochrome P450, retinol metabolism, bile secretion, PPAR signaling pathway, and carbon metabolism (Figure 7(b)).

### 3.5. Correlation Analysis between TTC36 Expression and Infiltrating Immune Cells

**Figure 8:**
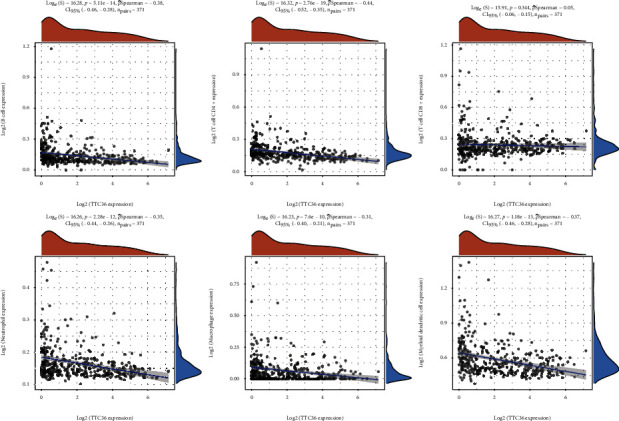
Correlation assays between TTC36 expressions and immune cell infiltration in HCC.

Growing evidences have confirmed that tumor-infiltrating lymphocytes may be involved in the long-term survival of patients with various tumors. Therefore, we examined the relationship of TTC36 expression with a variety of infiltrating immune cells. As shown in Figure 8, we observed that TTC36 expression has significant correlations with B cell, T cell CD4+, neutrophil, macrophage, and myeloid dendritic cell. These findings strongly suggest that TTC36 plays a specific role in immune infiltration in HCC.

### 3.6. Suppression of TTC36 May Reduce Proliferation and Invasion in HCC

**Figure 9:**
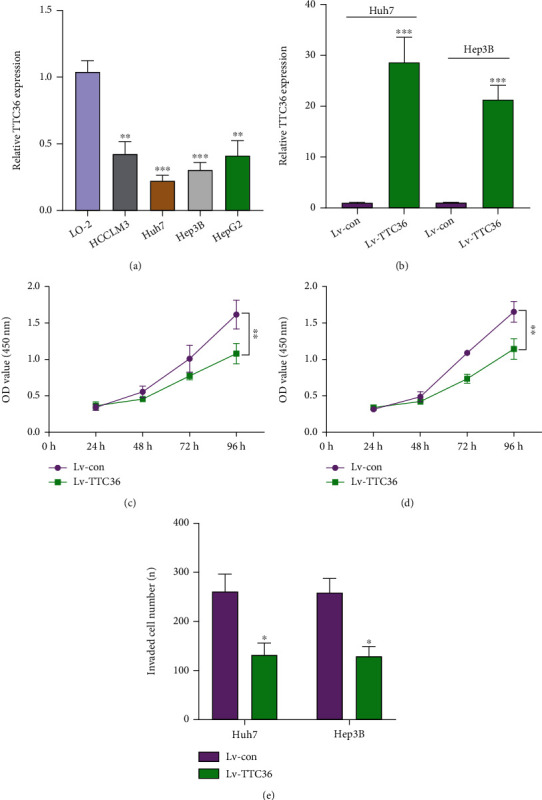
TTC36 inhibited the proliferation and invasion of Huh7 and Hep3B cells. (a) RT-PCR of TTC36 levels in HCC cells compared to L02 cells. (b) RT-PCR of TTC36 expression after lentivirus transfection. (c, d) CCK-8 was applied to determine the roles of TTC36 overexpression on proliferation of Huh7 and Hep3B cells. (e) Transwell assays were applied to detect the roles of TTC36 overexpression on invasion of Huh7 and Hep3B cells. ^∗^*P* < 0.05, ^∗∗^*P* < 0.01, and ^∗∗∗^*P* < 0.001.

To study the roles of TTC36 in HCC, we continued to research the function of TTC36 in cell proliferation and invasion. Firstly, we used the qRT-PCR to check the expression of TTC36 in HCC cell lines. It was said that TTC36 was lower expressed in HCCLM3, Huh7, Hep3B, and HepG2 compared with LO-2 (Figure 9(a)). Hep3B and Huh7 cell lines were infected with lentiviruses, and we overexpressed TTC36 using RT-qPCR to detect high overexpression efficiency (Figure 9(b)). Moreover, tests using CCK-8 showed that TTC36 overexpression slowed the growth of Hep3B and Huh7 cells (Figures 9(c) and 9(d)). Moreover, we tested the effect of TTC36 on HCC cell invasion. As shown in Figure 9(e), we found that Huh7 and Hep3B cell invasion was similarly considerably inhibited by overexpression of TTC36. These findings first showed that TTC36 is involved in limiting the growth and invasion of HCC cells.

## 4. Discussion

High molecular and cellular heterogeneity as well as a high rate of recurrence and metastasis makes HCC one of the most common and life-threatening tumors in the world [15]. Although surgical and medicinal treatment procedures have made rapid and significant improvement, the outlook for HCC patients remains bleak [16, 17]. The advancement of HCC is aided by the absence of early-stage detection indicators that are effective, and even among patients with the same TNM stage of cancer, survival periods vary substantially [18, 19]. Thus, novel accurate prognostic models and early diagnostic indicators are urgently needed in the diagnosis of HCC patients and the prediction of their survival.

In this study, we analyzed TCGA datasets and identified 35 DEGs between HCC specimens and normal liver specimens. Among 35 DEGs, our attention focused on TTC36. To date, the expression and function of TTC36 in tumors were rarely reported. Song et al. reported that when compared to nearby normal tissues, the expression of TTC36 in human gastric cancer tissues was much reduced, and this was demonstrated to be closely associated with clinical prognosis. Increased apoptosis in AGS cells was significantly reduced by the overexpression of TTC36 via Wnt-*β*-catenin pathway. This is the first study reporting the antioncogenic roles of TTC36 in tumor [20]. In the current investigation, we firstly reported that TTC36 expression was distinctly decreased in HCC specimens compared with nontumor specimens. Clinical assays revealed that in comparison to individuals with higher levels of TTC36 expression, patients with lower levels of the gene's expression had shorter overall life times. Importantly, TTC36 expression was found to be an independent predictive factor for overall survival in multivariate analysis results. On the other hand, we performed gain-of-function experiments and confirmed that overexpression of TTC36 distinctly suppressed the proliferative and invasive abilities of HCC cells. Our findings firstly reported the potential of TTC36 applied as novel prognostic biomarker for HCC patients.

Increasing amounts of evidence demonstrate that aberrant DNA methylation plays a crucial part in both the initiation and progression of HCC [21, 22]. Through the use of Pearson coefficients, we began our investigation by determining whether or not the level of methylation on TTC36 may affect the expression of TTC36 mRNA. In HCC tissues, there was a significant inverse relationship between the amount of methylation of TTC36 and the expression of TTC36 mRNA. This inverse association may very well explain why HCC tissues have such low levels of TTC36 expression. Then, we proceeded to identify the particular CpG sites in the TTC36 DNA promoter at which methylation was considerably linked with TTC36 expressions. Astonishingly, all the CpG sites, including cg01128850, cg03962678, cg16806210, cg19045070, and cg24222440, showed significant associations with TTC36 expression. Previous researches have shown that the link between the expression of a particular gene and its DNA methylation can range from being weak to being moderate, and limited genes have been shown to be substantially modulated by DNA methylation. Besides, we examined the importance of TTC36 DNA methylation in terms of prognosis. However, the results were not distinct. Overall, TTC36 was negatively modulated by TTC36 methylation, and the prognostic value of TTC36 methylation status needed to be further studied.

Recent years have seen a fast rise in attention to the tumor microenvironment (TME), which has been identified as a key determinant in tumor formation and progression, treatment resistance, and prognosis [23, 24]. According to most experts, HCC has a distinct immunological microenvironment. Several different types of immune cells have infiltrated the HCC. The presence of TICs in TME has been found in certain studies to be a promising predictor of outcome [25, 26]. The effectiveness of targeted therapy has significantly improved since the advent of immunotherapy. Combining VEGFR inhibitors with immune checkpoint inhibitors (ICIs) can improve patient tolerability and lengthen survival time in patients with HCC [27, 28]. In the current research, we examined the correlation of TTC36 expression with several kinds of infiltrating immune cells. We observed that TTC36 expression has significant correlations with B cell, T cell CD4+, neutrophil, macrophage, and myeloid dendritic cell. These findings strongly suggest that TTC36 plays a specific role in immune infiltration in HCC.

However, there were several limitations in this study. First, the sample size is relatively small; large clinical trials are needed to be conducted. Second, this is a study that was done in the past. Prospective studies are required to corroborate our findings because of the possibility of patient heterogeneity. Third, TTC36 and immunotherapy effectiveness in HCC patients can only be demonstrated in a limited way by our studies. More in vitro and in vivo experiments were needed to further confirm our findings.

## 5. Conclusion

For the first time, we have established conclusively in the present study that expression of TTC36 is downregulated in HCC and positively correlated with a low clinical stage. According to the findings of this investigation, TTC36 may be able to be identified as a potential biomarker linked with a bad prognosis and may have a specific function in immunological infiltration.

## Data Availability

The data used to support the findings of this study are available from the corresponding author upon request.
